# The interference of ozone gas in kinects and mitochondrial potential of equine sperm submitted on cryopreservation

**DOI:** 10.1590/1984-3143-AR2021-0075

**Published:** 2021-12-17

**Authors:** Iara Nóbrega Macêdo, Lucia Cristina Pereira Arruda, Breno Barros de Santana, Thalles Cloves Maciel de Moura, Maria Madalena Pessoa Guerra, Diogo Gutemberg Bezerra, Gustavo Ferrer Carneiro, Sildivane Valcácia Silva

**Affiliations:** 1 Programa de Pós-Graduação em Ciência Animal, Centro de Ciências Agrárias, Universidade Federal da Paraíba, Areia, PB, Brasil; 2 Departamento de Medicina Veterinária, Universidade Federal Rural de Pernambuco, Recife, PE, Brasil; 3 Central Monte Verde de Reprodução Equina, Sairé, PE, Brasil; 4 Centro de Biotecnologia, Universidade Federal da Paraíba, João Pessoa, PB, Brasil

**Keywords:** antioxidants, cryopreservation, ozone therapy, spermatozoa, stallion sperm

## Abstract

The objective of this study was to evaluate the effects of the addition of different concentrations of ozone to quarter horse semen submitted to cryopreservation. Six ejaculates from four stallions were collected and were divided in four experimental groups: a control group (BotuCRIO® extender) and three other groups with BotuCRIO® ozonized at concentrations of 6, 8 and 12 μg of O_3_/mL. The semen samples were diluted (200 x 10^6^ spermatozoa/mL), filled in straws and frozen. After thawing (37 ºC, 30s), the samples were evaluated at 0, 30 and 60 minutes of incubation regarding sperm kinetics by a computer-assisted sperm analysis (CASA), and plasma membrane integrity (PMI), acrosome integrity (ACi) and mitochondrial membrane potential (MMP) by fluorescent probes. There was a reduction in the kinetic parameters total motility (TM), progressive motility (PM), curvilinear velocity (VCL), straight line velocity (VSL) and average path velocity (VAP) in all groups during the thermoresistance test (TT), a pattern also found in PMI and MMP analyses (p<0.05). There was no difference (p>0.05) between the control and treatment (6, 8, and 12 μg of O_3_/mL) groups, in any of the evaluated times for the kinetic parameters TM, linearity (LIN), straightness (STR), wobble index (WOB), amplitude of lateral head displacement (ALH) and beat cross frequency (BCF). Regarding the VCL, VSL and VAP parameters, the group treated with 6 μg did not differ from the control or from 8 μg, but was higher than 12 μg at 30 and 60 minutes. ACi and PMI did not differ between groups (p>0.05), but PMI was lower in groups 8 μg and 12 μg compared to the control and 6 μg (p<0.05). It was concluded that the addition of ozone does not present beneficial effects for cryopreservation of equine semen at the concentrations used and decreases important parameters of fertility.

## Introduction

The cryopreservation of semen represents an important resource in the preservation of the equine species, both by the attempt to maximize fertility and by the use of genetically superior stallions. Associated with artificial insemination, the cryopreservation biotechnique allows the formation of a genetic bank of animals of high breed and commercial standard (Yimer et al., 2016) and the possibility of a stallion obtaining hundreds of descendants throughout its reproductive life (Canisso et al., 2008).

Although numerous studies are constantly developing and demonstrate advantages in its use, the frozen semen of stallions still presents variable fertility results (Santos et al., 2015). Research indicates that cryopreservation increases the production of reactive oxygen species (ROS) by spermatozoa (Lucio et al., 2016) and decreases antioxidant defenses present in semen (Martínez-Páramo et al., 2012).

With the objective of reducing oxidative damage due to the action of ROS, which can influence the sperm function and the imbalance between production and degradation (Bansal and Bilaspuri, 2010), researchers added antioxidants to the freezing extenders to improve post-thawing sperm viability (Li et al., 2018).

Ozone (O_3)_ is considered a potent oxidizing agent (Inal et al., 2011) that has aroused the interest of veterinary medicine and is being highlighted in the treatment of various pathologies (Greene et al., 1993; Hernández and González, 2001). It is a reliable, inexpensive therapy, with few adverse effects reported (Severo et al., 2019). It is produced by a medicinal generator, where, through high-voltage discharges, oxygen (O_2_) is converted to O_3_ (Smith et al., 2017).

In the bloodstream, O_3_ reacts immediately with oxygen and generates ROS and lipoperoxidation products (LOP); the body produces, as a response, therapeutic effects in varied cells (Mauro et al., 2019) and consequent bactericidal, anti-inflammatory, and analgesic effects (De Andrade et al., 2019). In addition, one of the biological effects is the stimulation of the antioxidant response of the body from endogenous-enzymatic mechanisms (Bocci et al., 2011).

Considering the need to minimize the deleterious effects caused by freezing/thawing of equine sperm, and the applicability of ozone therapy in veterinary medicine in order to stimulate a possible antioxidant response to the oxidative processes promoted by cryopreservation, the objective of the present study was to evaluate the addition of O_3_ to equine semen extender and its effect on sperm cell viability.

## Materials and methods

Prior to direct experimentation with animals, this project was submitted to and approved by the Ethics Commission on the Use of Animals (CEUA/UFPB) under protocol number 4277190820.

### Animals

Ejaculates of four quarter-horse stallions, (14.00 ± 8.76) years old and weighting (516.25 ± 28.10) kilograms, submitted to the same management (stabled animals, being provided 3.0 kg/animal/day of concentrate, Tifton hay, and water *ad libitum*), clinically healthy, with historic of fertility comproved and submitted to andrological examination, were used. The animals were from the Central Monte Verde de Reprodução Equina, located in Sairé, Pernambuco, Brazil (latitude: 8° 19’ 42” south, longitude: 35° 41’ 23” west). Semen samples were obtained in March and April of 2021.

### Semen collection and analysis

Six ejaculates were collected from each stallion, totaling 24 ejaculates, with harvests performed every other day. The ejaculates were obtained through the artificial vagina method (Botucatu model), with the aid of a mannequin. A collector cup composed of a plastic condom and a nylon filter for the removal of the gel fraction were located in the artificial vagina.

The ejaculates were immediately evaluated after harvest (CBRA, 2013). Macroscopic (volume, color, appearance, and odor) and microscopic analyses (motility and sperm vigor) were performed using the computerized analysis method (Mace Sperm Tracker, Anturius, Brazil) with a camera coupled to an optical microscope (BEL Tech Bio 2, BEL Engineering®, Italy).

During evaluation, the samples were kept on a heating plate at 37 °C. The sperm concentration was determined by a Neubauer chamber, using a ratio of 1:20 in distilled water, under optical microscopy in an increase of 40x (BEL Tech Bio 2, BEL Engineering®, Italy).

### Dilution and treatments

The ejaculate of each equine was diluted at a ratio of 1:1, in a commercial extender based on skimmed milk (BotuSemen®, Botupharma, Brazil), submitted to centrifugation (600 x g, for 10 minutes) for removal of seminal plasma. After centrifugation, the supernatant was removed and the pellet was resuspended in a commercial egg yolk extender (BotuCRIO®, Botupharma, Brazil), at a concentration of 200 x 10^6^ spermatozoa/mL. In the experimental group, the BotuCRIO® extender was ozonized through an ozone generator (Portable O&L Model, Ozone & Life®, São José dos Campos, Brazil) in a ratio of 1:1 of O_3_ volume and diluent volume, homogenized in syringe.

Each ejaculation, after centrifuging, was divided and added to four groups: a control group (BotuCRIO^®^, without adding O_3_ gas); BotuCRIO^®^ commercial extender, ozonized with 6 μg of O_3_/mL; BotuCRIO^®^ ozonized with 8 μg of O_3_/mL; and BotuCRIO^®^ ozonized with 12 μg Of O_3_/mL. Then, samples were filled into straws (0.5 mL), duly identified, sealed with polyvinyl alcohol, and submitted to cryopreservation.

### Cryopreservation

The cryopreservation process was carried out in a programmable freezing machine (TK 3000 CSE, TK Tecnologia em Congelação LTDA, Uberaba, MG, Brazil). Initially at room temperature (28 °C), the semen straws were subjected to a cooling curve (−0.5 °C/min) until reaching 5 °C and maintained at this temperature for 20 minutes (stabilization period). After this cooling period, the samples were submitted to a freezing curve (−15 °C/min) until reaching a temperature of −120 °C; then, they were immersed in liquid nitrogen and stored in cryogenic cylinders at −196 °C.

### Thawing and semen analysis

At the time of analysis, two straws from each experimental group were removed from the cryogenic canisters and thawed in a water bath at 37 °C/30 seconds. After that, they were submitted to the thermoresistance test (TT), considering M0 = immediately post-thawing, M30 = 30 minutes after thawing and incubation (37 °C), and M60 = after 60 minutes of thawing and incubation (37 °C). At each time, the sperm kinetics, PMI, ACi, and MMP were evaluated.

### Sperm kinetics

The following variables were analyzed through the computerized system of sperm analysis (CASA; SCA^TM^, Microptics, S.L. Version 5.1, Barcelona, Spain): total motility (TM; %), progressive motility (PM; %), straight line velocity (VSL; μm/s), curvilinear velocity (VCL; μm/s), average path velocity (VAP; μm/s), beat cross frequency (BCF; Hz), amplitude of lateral head displacement (ALH; μm), straightness (STR; %), linearity (LIN; %), and wobble index (WOB; %).

Aliquots of each sample (2.5 μL) of semen were evaluated individually on a slide covered with cover glass (18 x 18 mm), preheated at 37 °C, and examined under a phase contrast microscope (100x, Eclipse 50i, Nikon, Japan), with a 10x objective, coupled to the CASA system. At least 500 sperm were captured per sample, in five random, non-consecutive fields selected by the same operator.

The parameters of the CASA system were adjusted according to Nery et al. (2020), with the following settings: temperature of 37 °C; magnification 100 x; number of images per second 24; head area, 10–70 μm^2^; VAP: 10 μm/s slow, <45 μm/s medium, <90 μm/s fast; progressivity 75% STR; circular 50% LIN.

### Cellular health tests

The plasma membrane integrity tests and acrosome integrity, as well as the mitochondrial activity, were performed using fluorescent probes in epifluorescence 17 microscopy (Axiostar plus, Zeiss, Germany). Two hundred sperm cells were counted and 40x magnification was used for plasma membrane and mitochondria, and 100x for acrosome and were classified based on the fluorescence emitted from each probe (Silva et al., 2019).

All reagents were purchased from Sigma-Aldrich (St. Louis, MO, USA). Fluorophore stock solutions were prepared as follows: propidium iodide (PI, 25 mg/mL), JC-1 (5 mg/mL), and fluorescein isothiocyanate conjugated to peanut agglutinin (FITC-PNA). (FITC-PNA, 1 mg/mL) in phosphate buffered saline (PBS). The working solutions were JC-1 (153 µM) dimethyl sulfoxide (DMSO), FITC-PNA (0.04 mg/mL), PI (0.5 mg/mL) in PBS, and carboxyfluorescein diacetate (CFDA; 0.46 mg/mL in DMSO). All solutions were maintained at −20 °C until use.

Previously, plasma membrane integrity (PMI) and mitochondrial membrane potential (MMP) analyses were diluted in 200 μl of semen in TRIS-wash buffer (500 μL), centrifuged (100 x g/5 min) and resuspended in TRIS (60 μL); then, 30 μL was separated for evaluation of PMI and 30 μL for MMP.

PMI was evaluated by the double staining method of PI fluorophores and CFDA, detected by the inclusion of PI in the cell nucleus. A sample aliquot (30 μL) was stained with 5.0 μl of CFDA and 5.0 μl of PI and incubated for a period of 10 min at 25 °C. Sperm were evaluated using DBP excitation filters 485/20 nm and DBP 580–630 nm. Spermatozoa stained in green were considered intact and those stained in red were considered to have a damaged membrane.

The MMP was evaluated using a lipophilic cationic fluorophore (JC-1) associated with PI. The sample aliquot (30 μL) was stained with 5.0 μL of JC-1 and incubated for 10 min at 25 °C. Sperm were evaluated using BP 450–490 nm excitation and LP 515 emission filters. Spermatozoa with an intermediate part stained in orange were considered to have a high MMP, while sperm with a green intermediate part were considered to have a low MMP.

The acrosome integrity (ACi) assessment was performed by fluorescein isothiocyanate conjugated to peanut agglutinin (FITC-PNA). An aliquot (10 μL) of the sample was used to make a smear and air dried. The slides were stained with aliquots of 20 μl of FITC-PNA and incubated in a humid chamber at 4 °C for 15 min in the absence of light. Then, the slides were immersed in PBS twice and air dried. Immediately before evaluation, 5.0 μL of the solution (4.5 mL of glycerol, 0.5 mL of PBS, and 5.0 mg of p-phenylenediamine) were placed on the slide and covered with a cover glass. Sperm were evaluated using a BP 450–490 nm excitation filter and LP 515 nm emission. The acrosomes of sperm stained fluorescent green were considered to have an intact acrosome. When only the equatorial region of the sperm head was fluorescent green or when fluorescence was absent, sperm were considered to have a damaged acrosome.

### Statistical analysis

The statistical analyses were performed using the GraphPad InStat Software (version 3.10, 2009). The variables expressed in percentages were transformed by sine arc (sine arc √P/100). The data were initially submitted to a normality test (Kolmogorov–Smirnov) to identify the distribution of data and choose parametric or non-parametric tests. After identification, the data were submitted to variance analysis (ANOVA), followed by Tukey’s post-test if parametric, or Kruskal–Wallis if non-parametric. All tests described were performed with at least a 5% confidence level (p < 0.05).

## Results

Considering the absence of individual effect (p> 0.1), the ejaculates were grouped according to the experimental treatments. The samples were evaluated 0, 30, and 60 min after thawing. The kinetic parameters of post-thawing equine semen previously ozonated during cryopreservation are presented in Table 1. Independently of time (p > 0.1), an effect of treatment was not detected (p > 0.1) for LIN, STR, WOB, ALH, or BCF.

**Table 1:** Kinetic parameters (mean ± standard deviation) of equine semen cryopreserved with BotuCRIO® extender (control group) and BotuCRIO® added with ozone (6, 8 and 12 µg of O_3_ / mL), at 0, 30 and 60 minutes post thawing.

		**Control**	**6 µg O_3_/mL**	**8 µg O_3_/mL**	**12 µg O_3_/mL**
	**0h**	64.21 ±16.23^x^	67.13 ± 17.27^x^	61.46 ± 16.23^x^	60.40 ± 17.74^x^
TM	**30 min**	56.99 ± 16.37^xy^	57.98 ± 15.21^y^	57.98 ± 18.51^xy^	51.00 ± 18.00^y^
(%)	**60 min**	51.68 ± 19.30^y^	50.97 ± 16.37^y^	55.65 ± 19.67^y^	52.98 ± 19.77^xy^
	**0h**	30.30 ± 8.78^x^	31.03 ± 9.61^x^	27.88 ± 10.00^x^	26.68 ± 9.50^x^
PM	**30 min**	24.45 ± 8.35^ab.y^	27.58 ± 10.33^a.y^	24.90 ± 10.43^ab.xy^	21.17 ± 11.02^b.y^
(%)	**60 min**	21.25 ± 9.67^y^	21.93 ± 9.85^y^	21.78 ± 10.84^y^	19.48 ± 9.44^y^
	**0h**	84.95 ± 15.86^x^	84.57 ± 15.11^x^	80.50 ± 15.65^x^	78.32 ± 16.00^x^
VCL	**30 min**	76.06 ± 15.31^ab.y^	79.81 ± 15.65^a.xy^	73.83 ± 14.59^ab.xy^	69.03 ± 16.72^b.xy^
(μm/s)	**60 min**	72.33 ± 15.90^a.y^	71.13 ± 17.12^a.y^	67.21 ± 16.82^ab.y^	62.93±16.89^b.y^
	**0h**	48.33 ± 8.32^x^	48.14 ± 8.06^x^	45.34 ± 8.36^x^	44.47 ± 7.76^x^
VSL	**30 min**	42.85 ± 6.75^ab.xy^	46.93 ± 8.68^a.y^	42.42 ± 8.53^ab.xy^	39.64 ± 9.31^b. xy^
(μm/s)	**60 min**	40.32 ± 7.32^ab.y^	41.40 ± 9.18^a.y^	38.39 ± 9.17^ab.y^	35.65 ± 7.68^b.y^
	**0h**	62.71 ± 11.41^x^	62.45 ± 10.47^xy^	59.00 ± 10.86^x^	57.86 ± 10.65^x^
VAP	**30 min**	56.50 ± 10.57^ab.xy^	60.43 ± 11.44^a.x^	55.34 ± 10.61^ab.xy^	51.75 ± 12.44^b.xy^
(μm/s)	**60 min**	53.65 ± 11.40^a.y^	53.58 ± 12.21^a.y^	50.36 ± 12.32^ab.y^	46.65 ± 11.28^b.y^
	**0h**	57.43 ± 7.65	57.43 ± 6.40	56.77 ± 6.09	57.46 ± 6.13
LIN	**30 min**	57.18 ± 6.50	59.20 ± 4.71	57.63 ± 4.38	57.70 ± 6.44
(%)	**60 min**	56.31 ± 4.81	58.64 ± 6.15	57.38 ± 9.38	57.42 ± 7.12
	**0h**	77.28 ± 4.95	77.27 ± 4.57	76.98 ± 4.35	77.15 ± 4.25
STR	**30 min**	76.46 ± 5.66	77.89 ± 3.43	76.72 ± 3.48	76.76 ± 5.09
(%)	**60 min**	75.85 ± 5.52	77.54 ± 5.11	76.12 ± 6.79	76.84 ± 6.17
	**0h**	74.18 ± 6.77	74.22 ± 5.41	73.65 ± 5.25	74.40 ± 5.63
WOB	**30 min**	74.70 ± 5.62	75.96 ± 4.73	75.12 ± 4.42	75.03 ± 4.95
(%)	**60 min**	74.29 ± 4.02	75.54 ± 4.80	74.90 ± 7.73	74.52 ± 4.67
	**0h**	2.97 ± 0.53	2.98 ± 0.53	2.95 ± 0.45	2.90 ± 0.51
ALH	**30 min**	2.85 ± 0.45	2.78 ± 0.46	2.74 ± 0.48	2.65 ± 0.54
(μm)	**60 min**	2.66 ± 0.58	2.59 ± 0.71	2.52 ± 0.69	2.57 ± 0.67
	**0h**	11.32 ± 1.43	11.26 ± 1.57	11.17 ± 1.32	10.95 ± 1.39
BCF	**30 min**	10.90 ± 1.08	11.32 ± 1.31	10.53 ± 1.39	10.31 ± 1.99
(Hz)	**60 min**	10.54 ± 1.75	10.32 ± 2.50	10.10 ± 2.41	10.28 ± 2.52

(TM;%): Total motility; (PM; %): Progressive motility; (VSL; μm/s): straight line velocity; (VCL; μm/s): curvilinear velocity; (VAP; μm/s): average path velocity; (BCF; Hz): beat cross frequency; (ALH; μm): amplitude of lateral head displacement; (STR; %): straightness; (LIN; %): linearity; (WOB, %): wobble index. ^ab^ Lowercase letters on the same line represent difference between treatments (p<0.05); ^xy^ Lowercase letters in the same column represent time difference (p<0.05).

**Figure 1:**
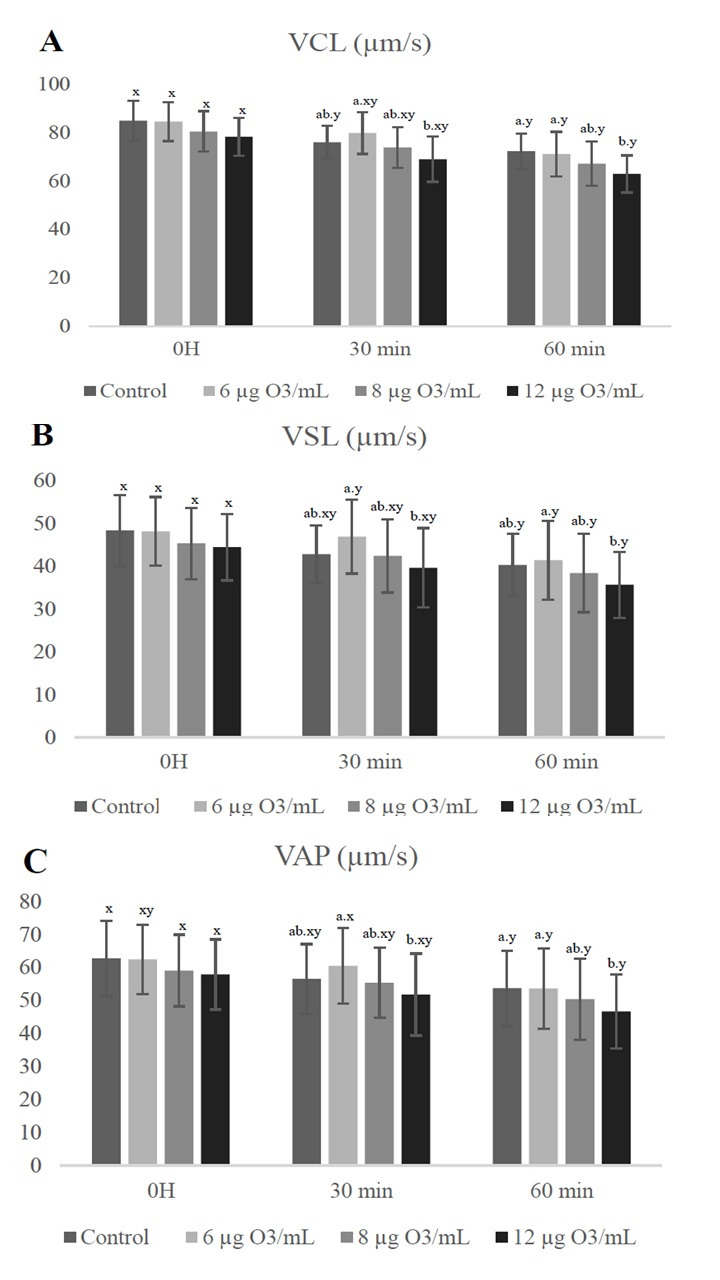
Speed values (mean ± standard deviation) of equine sperm with or without ozone at 0h, 30 and 60 min post thawing.

For VCL, VSL, and VAP, (Figure 1) there was no significant effect (p > 0.05) between the experimental groups at 0 h. At 30 min, for the three parameters, the group treated with 6 μg of O_3_/mL was higher (p < 0.05) than 12 μg O_3_/mL and did not differ (p > 0.05) from the control and 8 μg from O_3_/mL. At 1h of incubation, VCL and VAP of the control group and 6 μg of O_3_/mL, were higher (p < 0.05) than that of 12 μg of O_3_/mL and did not differ (p > 0.05) from 8 μg of O_3_/mL. For VSL, at 1 h, the 6 μg of O_3_/mL was higher (p < 0.05) than 12 μg of O_3_/mL and did not differ (p > 0.05) from the control and 8 μg of O_3_/mL.

Independently of group (p > 0.1), significant decreases (p < 0.05) of TM, PM, VCL, VSL, and VAP were detected throughout the complete incubation time. The results for PMI, ACi, and MMP are shown in Table 2.

**Table 2:** Plasma and acrosomal membrane integrity and mitochondrial membrane potential (mean ± standard deviation) measured by epifluorescence microscopy of equine semen cryopreserved with BotuCRIO® (control group) and BotuCRIO® plus ozone (6, 8 and 12 µg of O_3_/mL), evaluated at 0, 30 and 60 minutes post thawing.

		**Control**	**6 µg O_3_/mL**	**8 µg O_3_/mL**		**12 µg O_3_/mL**
	**0h**	40.13 ± 13.94^x^	43.85 ± 14.06^x^	43.77 ± 12.67^x^	39.98 ± 13.43^x^	
PMI	**30 min**	32.90 ± 11.91^y^	36.10 ± 9.93^xy^	38.19 ± 11.74^y^	33.31 ± 9.66^xy^	
(%)	**60 min**	26.85 ± 8.18^y^	30.33 ± 9.51^y^	30.38 ± 7.93^z^	28.31 ± 8.48^y^	
	**0h**	47.27 ± 14.77^a.x^	42.19 ± 14.23^ab.xy^	36.50 ± 15.01^bc. x^	29.49 ± 14.56^c^	
MMP	**30 min**	36.41 ± 15.71^a.y^	33.14 ± 17.39^ab.x^	30.96 ± 15.40^ab^	23.87 ± 13.19^b^	
(%)	**60 min**	26.22 ± 17.53^z^	23.96 ± 15.36^y^	23.43 ± 17.58^y^	16.36 ± 12.91	
	**0h**	25.46 ± 23.09	25.54 ± 26.16	23.00 ± 19.72	24.67 ± 24.19	
ACi	**30 min**	20.58 ± 19.99	22.71 ± 22.35	23.67 ± 23.53	19.46 ± 22.77	
(%)	**60 min**	18.92 ± 17.29	21.04 ± 20.09	20.46 ± 15.77	17.21 ± 15.90	

PMI: plasma membrane integrity; MMP: mitochondrial membrane potential; ACi: acrosome integrity. ^abc^ Lowercase letters on the same line represent difference between treatments (p<0.05); ^xyz^ Lowercase letters in the same column represent time difference (p<0.05).

**Figure 2:**
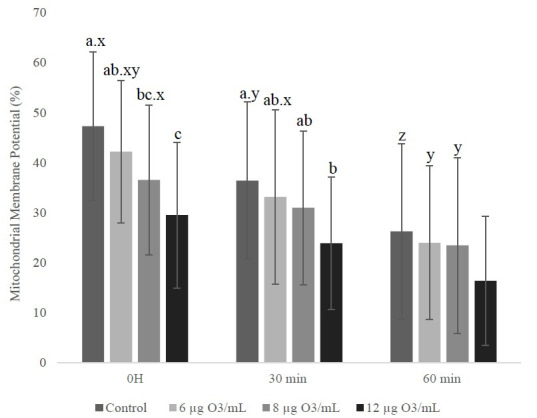
Percentage (mean ± standard deviation) of the mitochondrial membrane potential of equine sperm with or without ozone at 0h, 30 and 60 min post thawing.

There was no increase (p > 0.05) in the percentage of cells with intact plasma and acrosomal membrane integrity was not affected (p > 0.5) by the O_3_ treatment at any time. There was a reduction (p < 0.05) of cells presenting high MMP (Figure 2). The groups treated with 8 μg and 12 μg of O_3_/mL had lower MMP (p < 0.05), when compared to the control group at 0 h. The group with 12 μg of O_3_/mL was also lower (p < 0.05) when compared to 6 μg of O_3_/mL at 0 h. At 30 minutes, the 12 μg of O_3_/mL was lower (p < 0.05) than the control group. There was no difference (p > 0.05) among the groups at 60 minutes.

The percentage of cells with an integrated plasma membrane and with high MMP decreased (p < 0.05) over time in all groups, while the percentage of cells with intact acrosomes remained unaltered (p > 0.1).

## Discussion

In the present study, the effect of adding O_3_ to the cryopreservation diluent of equine semen on kinetics, PMI, ACi, and PMM immediately after thawing and for 30 and 60 minutes of incubation at 37 °C was evaluated. The TT was performed because it offers greater reliability in the measurement of parameters and indicators of fertility of sperm *in vitro* (Foster et al., 2011) and aims to simulate the permanence of sperm in the female’s genital tract (Arruda et al., 1992).

Therefore, it can be observed that there was a reduction in the parameters of TM, PM, VCL, VSL, and VAP in all groups throughout the TT, a pattern also found in the PMI and MMP analyses. Several authors are including the use of the TT to observe the longevity of post-thawing cells. Fürst et al. (2005), for example, also evaluated the longevity of thawed equine semen and observed, regardless of the protocol, that there was a decrease in sperm motility along the TT, as observed in the present study. One decrease in PMI after the TT may be justified by the damage caused to the structures and organelles involved in sperm movement during the freezing/thawing process (Watson, 1995 apud Santos et al., 2015).

Kinetic analyses also revealed no benefits in ozonizing the freezing extender in relation to the parameters of TM, LIN, STR, WOB, ALH, and BCF, which remained similarly preserved in all groups and times. These results complement the reports of Santos et al. (2018), that, when ozonizing the extender with 8 mg of O_3_/L and adding to fresh and chilled semen, did not observe differences in the percentages of TM, PM, and showed an intact plasma membrane in the two moments evaluated.

As for the values found for the velocities, it was observed that there was a negative correlation between a higher concentration of O_3_ and the velocities of VCL, VSL, and VAP, while lower concentrations were similar to the control action. These are important parameters in fertilization rates (Silva et al., 2017).

The lowest results found when using the concentration of 12 μg O_3_/mL proves that O_3_ is dependent dose, causing toxicity to cells at this concentration due to the greater amount of oxidation products generated. This also shows that the antioxidants present in the cell (minimum after removal of the seminal plasma) and in the extender were not able to balance the damage caused by oxidation.

PMI was not affected by added O_3_ in relation to the control group and treatments, which corroborates the studies by Santos et al. (2018), who demonstrated that adding semen to the ozonized extender did not alter the percentages of intact plasma.

In relation to MMP, there was a reduction of cells with high MMP and this occurred in treatments with higher concentrations of O_3_ in relation to the control group (8 and 12 μg of O_3_/mL). As we know, mitochondria are essential in sperm physiology and have their energy production generated by ATP synthesis, preceding oxidative phosphorylation, which enables sperm movement (Câmara and Guerra, 2008).

In summary, the excessive production of free radicals and ROS generated by O_3_, acts strongly on mitochondria, leading to mitochondrial impairment, with a reduction in ATP synthesis, a change in linear trajectory, and speed reduction, without necessarily increasing the amount of static spermatozoa (Ortega-Ferrusola et al., 2009), which would justify the changes found in the present study, where MMP, VCL, VSL, and VAP were reduced in treatments with higher concentrations.

Sperm have two sources of antioxidant defense, the enzymatic system, which has a limited defense capacity (Zini et al., 2000) and seminal plasma, which has extreme importance in protecting the cell against harmful effects of oxidative stress (Bucci et al., 2016). However, in this experiment, seminal plasma was removed in the centrifugation process, which could explain the ineffective protection from oxidation generated at greater concentrations of O_3_ in the results of this study.

It should be considered that the commercial extender usually includes antioxidants in its components, which shows us that these antioxidants may have been effective in controlling the oxidation caused at the concentration of 6 μg of O_3_/mL, as it did not present alterations in relation to the control group, or oxidation was not enough to damage the cell. Above 6 μg of O_3_/mL, there was a reduction of important fertilization parameters, with no benefits justifying the use of O_3_ in semen.

## Conclusion

Under these conditions, adding O_3_ gas to the equine semen freezing extender caused a post-thawing reduction in sperm kinetic parameters. Moreover, ozone therapy did not offer protective effects for the integrity of plasma and acrosome membranes. Finally, reduced MMP of equine sperm cells was associated with freezing with ozonated BotuCrio® extender.
